# Long-Term Results of stereotactic Brachytherapy (Temporary 125Iodine Seeds) for the Treatment of Low-Grade Astrocytoma (Grade II)

**DOI:** 10.5812/ircmj.4322

**Published:** 2013-01-05

**Authors:** Sohrab Shahzadi, Parisa Azimi, Khosrow Parsa

**Affiliations:** 1 Department of Neurosurgery, Shahid-Beheshti University of Medical Science, Tehran, IR Iran; 2 Department of Neurosurgery, Firozgar Hospital, Tehran, IR Iran

**Keywords:** Survival, Long-Term, Astrocytoma, Brachytherapy

## Abstract

**Background:**

Treatment of low-grade astrocytoma (WHO grade II) (LGA II) remains a challenge. There is limited information regarding the long-term effects of stereotactic brachytherapy (SBT) (temporary ^125^Iodine seeds) on patients with LGA II.

**Objectives:**

The purpose of this study was to evaluate disease control and survival after stereotactic brachytherapy in patients with circumscribed and relatively small size tumors.

**Materials and Methods:**

A retrospective review of 29 patients, treated between 1991 and 2011, was conducted to evaluate survival, complications, and local disease control after stereotactic brachytherapy. They belonged to a larger group of 48 cases with low-grade gliomas, treated with stereotactic brachytherapy. The demographic and clinical characteristics in patients including age, sex, and survival time were extracted from records.

**Results:**

Thirteen patients were male and 16 were female, with the median age of 29 years (range, 2.5 – 64 years). The median follow-up was 95 (range, 6 – 240) months. Based on Pignatti classification, 10 patients were at low- and 19 patients at high-risk. The median overall as well as progression-free survivals for patients were 135 months (95% confidence interval: 76 – 194) and 96 months (95% confidence interval: 1 – 199), respectively. Five- and 10-year progression-free survivals were 41.4 % and 34.5 %, respectively, and the 5- and 10-year overall survivals were 65.5 % and 44.8%, respectively. Progression-free survival was not significantly higher in smaller size tumors (P = 0.224), nor for spherical versus non-spherical tumors (P = 0.307). There was no treatment-related morbidity after stereotactic brachytherapy, and no radiogenic complications occurred during the follow-up period. Mortality due to tumor progression occurred in 4 patients (14%), and 11 patients were alive at the last follow-up.

**Conclusions:**

The stereotactic brachytherapy for patients with circumscribed and relatively small size tumors appears to be a safe, feasible, and minimally-invasive treatment.

## 1. Background

Low-grade gliomas (LGGs) (World Health Organization grade I and II) are slow growing brain tumors which constitute about one-sixth of glioma brain tumors (1). According to the World Health Organization (WHO) data, low-grade astrocytomas (LGAs II) (Grade II) comprise 15 to 25% of LGGs, constituting only 8% of recorded astrocytomas. For most patients with LGAs, there is no clear causal factor, and the current research indicates that the environment might play a role in their origin. Although LGA II was traditionally considered as benign, it could behave aggressively and undergo anaplastic transformation within 5 years in approximately half of the patients (2, 3). Malignant transformation occurs in 36–86% (4) of LGG patients, in 90% of adults (5, 6), and less than 10% of children with LGA II (7). Pignatti and colleagues ^8^ have classified LGG patients based on 5 criteria: age ≥ 40 years, astrocytoma histology, largest dimension of tumor ≥ 6 cm, tumor crossing the midline, and the presence of neurologic deficit before resection which are unfavorable prognostic factors (8). Patients with up to two of these findings are considered as low-risk, while patients with three or more are identified as high-risk (8).

Management, treatment, and survival of patients with LGA II are one of the challenges for a neurosurgeon (9). Different treatment options are available for LGA II at the present. Although surgery, radiation therapy (RT), stereotactic radiosurgery (SRS), and chemotherapy might be used to treat LGG, either separately or in combination (9), intraoperative implantation of ^125^Iodine seeds for brachytherapy (i.e. interstitial and intracystic) is also possible (10). Brachytherapy has a long history in the treatment of central nervous system tumors with the first treatment being carried out by Hirsch et al. in 1912 (11), and stereotactic (interstitial) ^125^Iodine was first used in brachytherapy of LGGs, in 1984 by Gutin et al. (9) Using temporary or permanent implants yet remain controversial (9). While some investigators advocate using permanent seeds (12), others prefer to use temporary implants (13).

Stereotactically guided interstitial therapy is a treatment option for gliomas (14). Modern imaging systems for tumor localization (15, 16) and three-dimensional radiation treatment planning programs to determine optimal access points and management of dose distribution (9, 17) have the potential to improve the stereotactic brachytherapy (SBT) results. The present retrospective study reports our experience with stereotactic (interstitial) ^125^Iodine brachytherapy (SBT) in patients with LGA II. Survival, complications, tumor control, and 20 years outcomes among our patients are evaluated.

## 2. Objectives

The purpose of this study was to evaluate disease control and survival after stereotactic brachytherapy for patients with circumscribed and relatively small size tumors.

## 3. Materials and Methods

### 3.1. Patients and Data Collection

**Figure 1:**
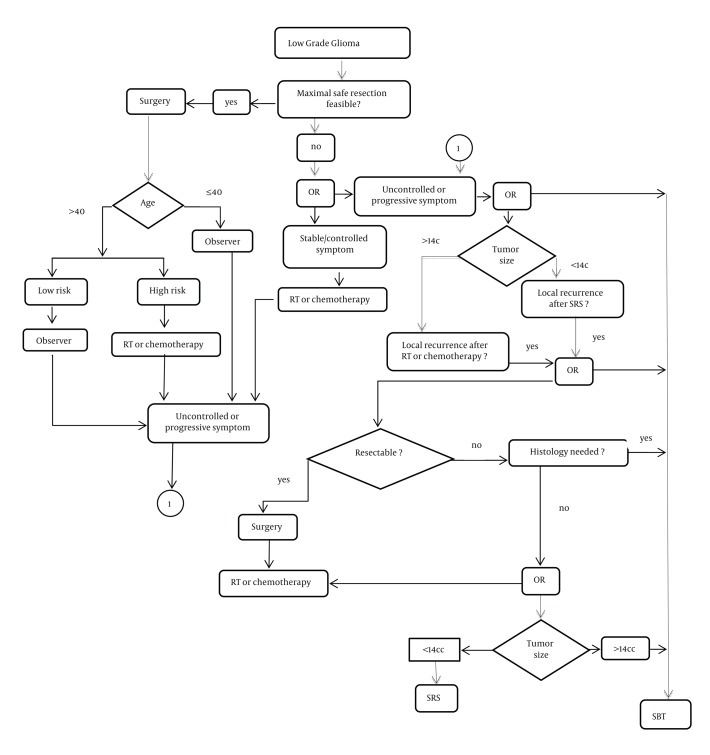
The algorithm shows a decision process for treatment selection in low-grade gliomas. SBT, stereotactic (Interstitial) ^125^Iodine brachytherapy; SRS, Stereotactic Radiosurgery; RT, Radiation Therapy

During a 20-year (January 1991-January 2011) period, 48 patients with circumscribed and relatively small size LGGs were treated in our department with stereotactic implantation of ^125^Iodine seeds, of which 29 were eligible to enter this study (patients with astrocytoma grade 2). Cases with oligodendroglioma, mixed glioma, and astrocytomas (all with Grade I) were excluded to avoid selection bias. Patients were selected for SBT protocol if they fulfilled at least one of the criteria listed in Figure 1 . The criteria were set based on previous experience in our clinic and National Comprehensive Cancer Network Clinical Practice Guidelines in Oncology (18). In summary, patients were treated with SBT if: A. Maximal safe resection of their tumor was not feasible; B. Their tumor’s histology was needed; and C. Local recurrence occurred after treatment with SRS, RT, or surgery. Eleven patients (38 %) had undergone surgery with partial resection before SBT, and in 18 cases (62%), maximal safe resection was not feasible. In 15 out of 29 cases (51.7%), SBT was offered primarily because a stereotactic biopsy was necessary. Additional selection criteria in favor of SBT and against SRS included tumor volume ≥ 14cc in 8 patients (27%), and local tumor recurrence after SRS and/or RT in 3 patients (10%).

The risk assessment for patients was based on classification by Pignatti and colleagues (8). Interstitial temporary SBT was performed utilizing photon radiation of ^125^Iodine seeds (model 6702, Medi-Physics Inc., Amersham Health Care, USA, measuring 4.5 × 0.8 mm, photon energy spectrum 27 – 35 keV).

### 3.2. Treatment Planning and Surgical Procedure

**Figure 2:**
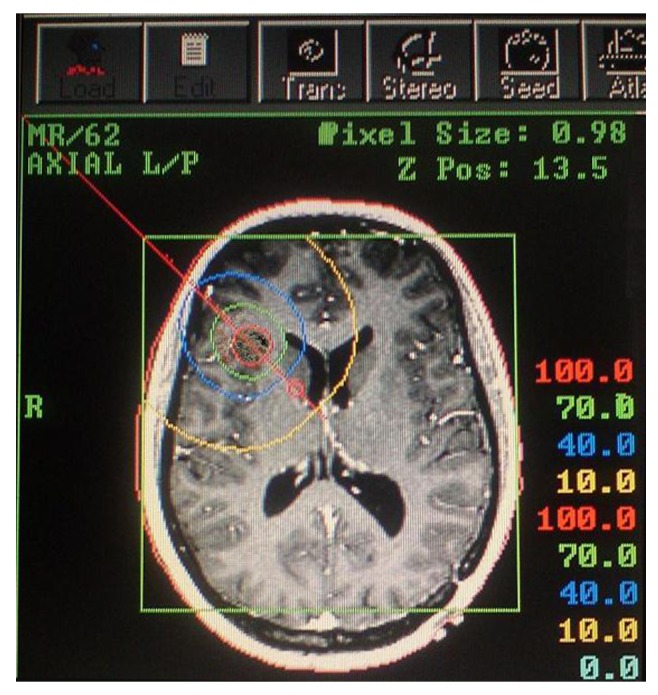
An Implant Planning for a Right Frontal Low-Grade Glioma Shows the Catheter Trajectory

Access and dose planning were performed on the basis of CT images (2 mm slice thickness, 290 mm field of view (FOV), 512 matrix) with a stereotactic localizer (19). Using Stereotactic Treatment Planning “STP” (Fischer Leibinger, Freiburg, Germany) (20), a 3D multiplanar treatment planning system, a protocol was designed to deliver optimum dose to the target volume (Figure 2) . The specified doses corresponded to the TG43 protocol (21). Prescribed cumulative reference dose on the surface of tumor was 60 Gy. The median D90 (defined by the dose that covers 90% of the tumor volume) and the V150 were 57.2 Gy (range: 44.3 - 79.2 Gy) and 57.8%, respectively. All patients were operated on using ^125^Iodine seeds placed in standard catheters under general anaesthesia, with the patient's head fixed in a modified Riechert – Mundinger (RM) stereotaxic frame (19). Stereotactic ^125^Iodine seed implantation was performed immediately after histological confirmation of LGG in biopsy. The correct positioning of the seeds was confirmed on post-implant CT or MR data sets by fusion with the treatment plan. In most cases, one seed was inserted, but two seeds were implanted in 7 patients in whom the sizes and shapes of tumors made it necessary based on treatment planning (TP) mentioned above. Biopsies were taken along a trajectory representative of the tumor at least one time before and after the procedure as needed. The average amount of tissue per biopsy specimen was 1 mm^3^. Hospitalization time was usually 3 – 4 days for biopsy and seed implantation, and one more day to remove the seed catheter(s).

### 3.3. Follow-up

The reference points for this study were the date of initial SBT procedure. The primary end-points for the statistical analysis were overall survival (OS) which was defined as the time from study entry to death from any cause; progression-free survival (PFS) was defined as the minimum time from study entry to disease progression, disease relapse, or death from any cause. The follow-up period for all patients was 6 months to 20 years during which the PFS and OS for all patients were recorded. Side effects and changes in imaging after SBT were also observed during the follow-up period.

### 3.4. Statistical Analysis

Kaplan–Meier survival curves were generated for each study variable using PASW Statistics 18, version 18 (SPSS, Inc., 2009, Chicago, IL). Survival rates for 5, 10, and 20 years were calculated along with their corresponding standard error (SE). Data are given as mean, median, and SD. Values of P ≤ 0.05 were considered statistically significant.

### 3.5. Ethics

All patients gave their informed consent after receiving both written and oral information about the project. The study was approved by the Ethics Committee of Shahid Beheshti University of Medical Sciences, Tehran, Iran.

## 4. Results

Patient characteristics and outcomes are summarized in Table 1 and Table 2, respectively.

**Table 1:** Patient Characteristics (n = 29)

	No. of Patients	Percentage (%)	Median ( ± SD), range
**Age, years**			29 ± 15.6 (2.5 - 64) years
**Gender (Female/Male)**	16/13	55/45	
**Follow up time**			95 ± 77 (6 - 240) months
**KPS Preoperative**			93.45 ± 9.36 (70 - 100)
**Previous surgery**	11	28.9	
**Previous chemotherapy**	0	0	
**Previous radiotherapy**	2	6.9	
**Previous radiosurgery**	1	3.4	
**Neurological Symptoms (Yes/No)**	18/11	62/38	
**High/low-risk patients**	10/19	35/65	
**Tumor site**			
**Central (hypothalamic/thalamic/optic)**	10	35	
**Cerebral hemisphere**	16	55	
**Posterior fossa**	3	10	
**Histologic type at diagnosis**			
**Astrocytoma II**	29	100	
**Tumor shape**			
**Spherical/non-spherical**	19/10	65/35	
**Tumor volume (cc)**			13.9 ± 8.8 (4.2 - 33.6) cc
**Source dwell time**			97 ± 24.9 (20 – 120) days
**Energy dose rate**			8.32 ± 1.58cGy/h
**Dosage at the tumor margin**			60 ± 1.03 (50 – 60) Gy
**Activity of each seed at implantation time**			9.2 ± 11.2 (5 - 16) mci

**Table 2:** Patient Outcomes (n = 29)

	No. of Patients	Percentage (%)	Median ( ± SD), range
**KPS at last follow-up**			95.52 ± 10.2 (80 - 100)
**Tumor Volume at Final Follow-up**			
**Increase/Stable or decrease**	6/23	21/79	
**Survival**			
**Overall survival(OS)**			135 (76 – 194) months
**Progression free survival(PFS)**			96 (1 - 199) months
**Cause of death**			
**Neurological death**	3	10	
**Systemic death**	12	41	
**Information missing**	3	10	
**Living patients**	11	39	

### 4.1. Survival

**Figure 3:**
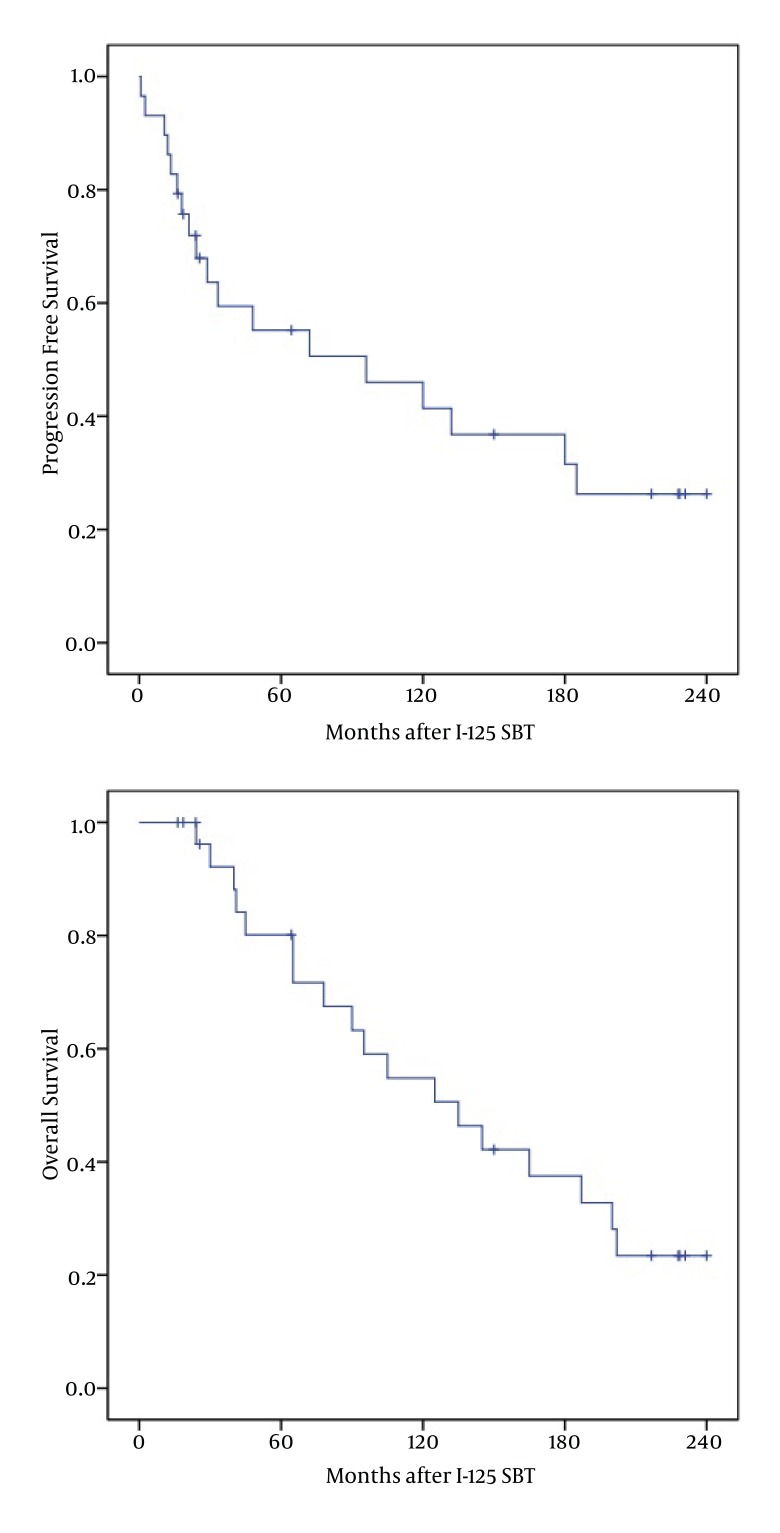
Kaplan-Meier Survival Curves for Patients Treated with Stereotactic ^125^Iodine Brachytherapy for Low Grade Astrocytoma Grade II. A. Progression Free Survival; B. Overall Survival. Median OS and FPS was 135 and 96 Months, Respectively

**Figure 4:**
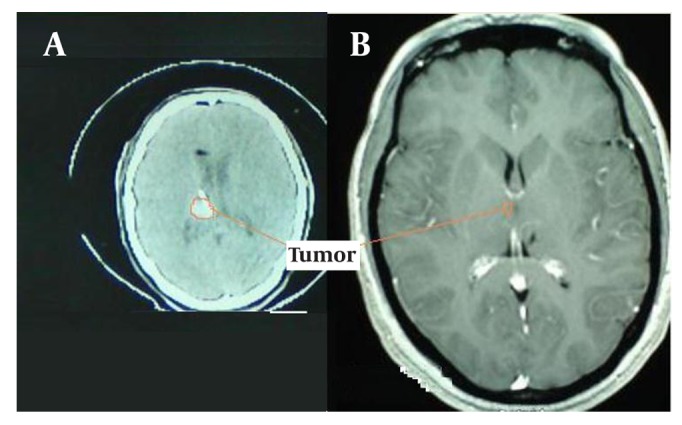
A Preoperative CT Scan from One Patient Undergoing SBT Treatment (B). Postoperative MRI from the Same Patient Nineteen Years Later (A)

The median FPS and OS for patients was 96 months (95% confidence interval: 1 – 199), and 135 months (95% confidence interval: 76 – 194) ( Figure 3 , A and B ), respectively. Five- year (10 - year) PFS was 56 % (47 %), and 5-year (10 - year) OS was 81 % (56%), respectively. Progression-free survival was not significantly higher for smaller tumor (P = 0.224), nor for spherical versus non-spherical tumors (P = 0.307). Of 11 patients who were alive at the last follow-up, no evidence of tumor recurrence and radiation encephalopathy was observed, and 4 (14%) patients survived over 17 years. No significant improvement in 5-year PFS or 5-year OS was seen when comparing patients receiving preoperative RT with those with no preoperative RT. A preoperative CT scan from one patient undergoing SBT treatment and the postoperative MRI from the same patient nineteen years later is present in Figure 4.

At the time of analysis, 11 out of 29 patients (38%) were alive and well: 6 patients had SBT as primary treatment, 3 had SBT after a surgical resection, and 2 had SBT for a recurrence after previous external beam RT. Four patients (14%) died from tumor progression: three died from neurological complications, and one from systemic complications associated with tumor recurrence. Eleven patients died with other non-tumor related systemic causes. Only four patients died due to the complications of tumor progression in our study; so, no statistical comparison of survival based on different methods was possible. 

### 4.2. Local Tumor Control

A local tumor control rate of 86% was achieved at last follow-up. Local growth of tumor after SBT was observed in 2 cases at 13 and 28 months, and SBT was repeated.

### 4.3. Complications

No serious neurological complications followed the SBT, and the quality of life was not seriously affected by the treatment in other patients. In 10 out of 11 patients who were alive, function was estimated to be good or excellent based on Karnofsky performance status (KPS). In one patient with poor function, the cause was found to be both treatment- and disease-related.

## 5. Discussion

To our knowledge, several studies have reported the long-term outcome of SBT and other treatment options for LGGs in the English literature (Table 1) (14, 22-31). Our findings were promising in treating patients with circumscribed and relatively small size LGGs. However, the favorable results could possibly be explained by selection of patients with well-established favorable prognostic factors such as younger age (mean: 29 years), almost perfect clinical condition (KPS mean: 93.45), and small tumors (mean: 13.9 cc). These patients might probably do equally well with external beam radiotherapy alone or in combination with surgery (8, 24, 27). The five-year survival rates of brachytherapy using SBT method have been reported to be between 54.6% to 97% (14, 22, 23, 30, 31), which is in line with our findings. A direct comparison between the outcomes of different methods used by us and other studies might be misleading, as the baseline characteristics of patients are different. As an example, the better outcomes reported by Korinthenberg et al. (31) and Wisoff et al. (28), might be due to the presence of younger patients in their study compared to our study. In a study conducted by Ahmadi et al. (25), only 46% of patients had pathology of astrocytoma, which might contribute to better 5 and 10 years survival rates reported by them. In the study by Kevin et al., only 7% of patients had grade II astrocytoma and 55% had grade I with a mean age of 8.7 years for patients, which might again contribute to their good reported results. Bauman et al. (26) reported a long-term (20 years) treatment of supratentorial LGGs with surgery, RT and chemotherapy, either separately or in combination, with a median follow-up of 105 months. The median PFS was 61 months (95% confidence interval: 53–77), and the median OS was 118 months (95% confidence interval: 93 – 129). In a retrospective analysis of 250 patients with WHO grade II astrocytomas treated with either permanent or temporary ^125^Iodine implants, Kreth et al. (22) reported a 5-year survival rate of 61%. This study used both temporary and permanent implants. Using temporary or permanent implants are one of the controversies of brachytherapy application in central nervous system tumors (9). However, better outcomes have been reported by Schnell et al. (14). It has been suggested that these findings might be related to the effects of selection, the relatively short follow-up period, and/or the benefit of combined treatment approach (14).

In the subsequent study, Kreth et al. (32) also suggested the SBT option to be associated with prolonged survival (10-year survival rate in the range of 84% and low-risk in patients with small tumor volumes). Another study by Central Brain Tumor Registry of the United States (CBTRUS) reported that grade II oligodendrogliomas had much better 5-year survival rate (70%) compared to mixed gliomas (56%) and astrocytomas (37%) (33). Another factor to consider while comparing outcomes is the fact that patients with more superficial tumors have undergone radiotherapy, and patients with more deeply seated tumors have been candidates for SBT method. At the time of analysis, 11 out of 29 patients (38%) were alive and well: 6 patients had SBT as primary treatment, 3 had SBT after a surgical resection, and 2 had SBT for a recurrence after previous external beam RT. The other information is provided in Table 3. Due to limited number of patients with prior radiotherapy, we could not compare these survival rates.

**Table 3:** Comparison of Study - Results with Those in Literature

Author (ref.)	Year	LGGs	No. of Patients	Treatment	SBT		5/10 - year	5/10 - year	15/20 - year	15/20 - year
					Temporary	Permanent				
**Kreth et al. (22)**	1995	Astrocytomas (II)	250	SBT	+	+	OS (%)	PFS (%)	OS (%)	PFS (%)
**Mehrkens et al. (23)**	2004	Astrocytomas of the Insula of Reil (II)	55	SBT	+	+	61/51	NR	NR	NR
**Van den et al. (24)**	2006	LGGs (II)	157	RT	-	-	54.6 /28.4	40.7 /20.2	NR	NR
**Schnell et al. (14)**	2008	LGGs (II)	31	S + SBT	+	-	66/ NR	35/ NR	NR	NR
**Ahmadi et al. (25)**	2009	Supratentorial LGGs (II)	130	S + Adjuvant treatment	-	-	93/ NR	62/ NR	NR	NR
**Bauman, et al. (26)**	2009	Supratentorial LGGs (II)	145	RT or S or Ch, either separately or in combination	-	-	85/55	41/11	36/28	0/0
**Schomas et al. (27)**	2009	LGGs (II)	314	RT or S or Ch, either separately or in combination	-	-	NR /48	NR /18	NR /22	NR /0
**Wisoff et al. (28)**	2011	LGG (I,II)	518	S + Adjuvant treatment	-	-	58/36	50/27	23/17	17/15
**Kevin et al. (29)**	2011	LGG (I,II)	181	RT or S or Ch, either separately or in combination	-	-	97/95	79/73	NR	NR
**Suchorska et al. (30)**	2011	LGGs (II)	95	SBT after S.	+	+	95/90	70/65	81/NR	60/NR
**Korinthenberg et al. (31)**	2011	LGGs (II)	94	SBT	+	-	NR	43.4/10.7	NR	NR
**Present study**	2011	Astrocytoma (II)	29	After RT or S, either separately or in combination, SBT performed	+	-	97/92	59/38	NR	NR

The OS and PFS of LGA II patients with astrocytoma histology are worse than the rest of the LGG patients (34). In LGG patients, the factors consistently known to be associated with improved survival include younger age (34, 35), higher KPS score (36), pathology (34), size of tumor (34, 37), postoperative RT, (17) tumor location (14, 38) (eloquent areas and deep-seated lesions are worse than other areas), and gross total resection (GTR) (25, 38). In the present study, we were not able to show any difference in OS or PFS based on these factors. The indiscrimination might be related to the small number of patients, or to the fact that all patients received SBT, after entry to the study and all had uniform astrocytoma (grade II) histology. Tacke et al. (28) reported the incidence of vasculopathy in children with hypothalamic/chiasmatic gliomas treated with SBT, but this complication was not observed in our study. Older reports have found a mortality rate of 3.3% to 6.5% after surgical resection of low-grade astrocytomas, but the benefit of radical surgical resection in the survival of patients with astrocytomas remained controversial (39). In a recent report by Chang et al. (40), the survival rate in the group undergoing surgery for LGG in eloquent areas of the brain using mapping – guided resection have been reported for 5-year OS and 5-year PFS as 86% and 62%, respectively, which demonstrated better outcomes when more modern surgical techniques were used.

In our study, the mortality due to tumor progression occurred in 4 patients (14%). The minimally invasive nature of SBT and the overall postoperative status in our cases would indicate that quality of life was not seriously affected by the treatment. During the analysis, 18 out of 29 patients (62%) died: 9 patients had SBT as primary treatment, 1 had SBT after a SRS, and 8 had SBT for a recurrence after previous surgical resection. There was no sufficient data available to compare outcome measurements after SRS with those after brachytherapy. Considering the fact that a proper guideline was not suggested to choose a treatment method for these patients, we tried to devise such a guideline (Figure 1). We feel that because of our study limitations, this guideline is still primitive and needs to be re-evaluated and modified based on future research efforts. For example, we feel a need to separate guidelines for children and adults, but our data were not suitable to devise.

### 5.1. Limitations and strength of the present study

There are several principle weaknesses in this study. The first is retrospective nature and inherent limitations of this methodology. Second is the comparatively small number of patients. Although, this is the largest single-institution series of LGA II reported in 29 patients treated with SBT in Iran, it remains a small number for statistical comparison. Our study also suffers from some other limitations like different treatments for patients prior to entering our study, and the variation in tumor location.

Finally, due to different patients’ baseline characteristics, it is very difficult to compare these results with that of primary surgery, with or without external beam radiotherapy, or with more conventional treatments of recurrent LGG. Even compared to other brain tumors, SBT series needs some reservation. However, the current study had several strengths including a 20-year fallow-up survival analysis, using temporary ^125^Iodine seed as SBT, and providing a flow chart for the first time as a guideline for SBT.

The SBT for patients with circumscribed and relatively small size tumors appears to be a safe, feasible, and minimally-invasive treatment.
